# Towards the optimization of passive undulatory locomotion on land: mathematical and physical models

**DOI:** 10.1098/rsif.2023.0330

**Published:** 2023-08-09

**Authors:** Basit Yaqoob, Emanuela Del Dottore, Alessio Mondini, Andrea Rodella, Barbara Mazzolai, Nicola M. Pugno

**Affiliations:** ^1^ Laboratory for Bioinspired, Bionic, Nano, Meta Materials and Mechanics, Department of Civil, Environmental and Mechanical Engineering, University of Trento, Trento 38122, Italy; ^2^ Laboratory of Bioinspired Soft Robotics, Center for Convergent Technologies, Istituto Italiano di Tecnologia, Genova 16163, Italy; ^3^ Department of Structural and Geotechnical Engineering, Sapienza University of Rome, Rome 00184, Italy; ^4^ School of Engineering and Materials Science, Queen Mary University of London, London E1 4NS, UK

**Keywords:** undulatory locomotion, passive adaptability, endogenous and exogenous dynamics, stiffness distribution, speed maximization

## Abstract

The current study investigates the body–environment interaction and exploits the passive viscoelastic properties of the body to perform undulatory locomotion. The investigations are carried out using a mathematical model based on a dry frictional environment, and the results are compared with the performance obtained using a physical model. The physical robot is a wheel-based modular system with flexible joints moving on different substrates. The influence of the spatial distribution of body stiffness on speed performance is also investigated. Our results suggest that the environment affects the performance of undulatory locomotion based on the distribution of body stiffness. While stiffness may vary with the environment, we have established a qualitative constitutive law that holds across environments. Specifically, we expect the stiffness distribution to exhibit either an ascending–descending or an ascending–plateau pattern along the length of the object, from head to tail. Furthermore, undulatory locomotion showed sensitivity to contact mechanics: solid–solid or solid–viscoelastic contact produced different locomotion kinematics. Our results elucidate how terrestrial limbless animals achieve undulatory locomotion performance by exploiting the passive properties of the environment and the body. Application of the results obtained may lead to better performing long-segmented robots that exploit the suitability of passive body dynamics and the properties of the environment in which they need to move.

## 1. Introduction

In limbless animals, lateral undulatory locomotion is the most common paradigm, in which the body bends laterally in a sinusoidal shape. This type of locomotion has long attracted the interest of scientists from the perspectives of evolutionary biology [1,2], physiology [3–5], morphology [6–8] and mechanics [9,10]. Several physical models of undulatory robots have been developed, inspired by snakes [11–14], salamanders [15], centipedes [16] and *Caenorhabditis elegans* [17], in order to demonstrate and understand different concepts involved in undulatory locomotion.

In the complex body of animals, active and passive mechanics play a critical role. In undulatory locomotion, the role of passive dynamics has been little studied compared to active dynamics. Incorporating passive dynamics through materials and morphology can lead to energy efficient, sustainable, robust, easy to control, self-adaptive and safe systems [18]. In [19], passive properties of the body are shown to help snakes manoeuvre through heterogeneous environments with minimal sensing. Furthermore, the passive stiffness of lamprey tail is investigated to generate different wake structures for different stiffnesses [20].

While the interaction of an animal body with its environment, exogenous effects, has been extensively studied in fluidic environments [10,20–23], little is known about the passive adaptability of undulatory locomotion on land. In [9], undulatory locomotion endogenous, characterized by body properties and kinematics, and exogenous effects in a dry frictional environment are investigated. The authors modelled undulatory locomotion in an isotropic frictional environment and suggested that endogenous parameters do not play a significant role in gait modulation. However, these results have not been validated on the physical system. Wang & Alben simulated the sinusoidal heaving of a thin, flexible foil at one end in an anisotropic dry frictional environment [24]. Findings give important insights into the role of resonance on the input power and speed of undulatory locomotion. Power increases and speed decreases at resonance in a low-frictional anisotropic environment; however, both speed and power vary smoothly in a high-frictional anisotropic environment. Some studies have modelled undulatory locomotion in granular media [25,26]. In [25], simulations are accompanied by a physical validation. The authors compared swimming speeds and forces obtained from simulated and physical models, demonstrating the importance of head drag on swimming speed and energy consumption. However, further investigation of the interconnection between endogenous and exogenous parameters and their effects on the dynamics of lateral undulatory locomotion is required. Therefore, in the present paper we endeavoured to correlate passive endogenous and exogenous effects of lateral undulatory locomotion for speed optimization. We will explore how exogenous effects, generated during body–environment interaction, and endogenous effects, generated by the inherent body stiffness and internal losses, influence the trajectory and the system speed. The results obtained from the mathematical model and physical system will be compared. Furthermore, we will suggest how to define the optimal body stiffness distribution to maximize the locomotion speed in relationship with specific environments. It should be noted that the objective of our study is not to replicate the snake-like lateral undulatory locomotion but rather to investigate and analyse the functional aspects of lateral undulatory locomotion involved in passive compliance using mathematical and physical models. Therefore, the physical model is designed accordingly, and functional aspects of endogenous and exogenous parameters are discussed in correspondence to the locomotion of animals.

## 2. Material and methods

### 2.1 Mathematical modelling

**Figure 1.**
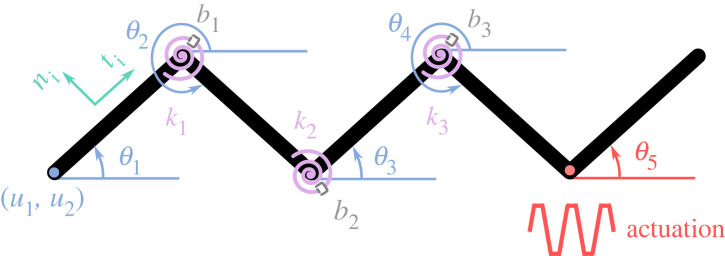
Schematic of the body discretized into five links. *k*_1_, *k*_2_ and *k*_3_ represent the rotational stiffnesses of the joints 1, 2, and 3, respectively. *b*_1_, *b*_2_, and *b*_3_ represent damping of the joints 1, 2 and 3, respectively. *θ*_1_, *θ*_2_, *θ*_3_, *θ*_4_, and *θ*_5_ represent link angles of links 1, 2, 3, 4 and 5, respectively. Link angle 5 is actuated upon a trapezoidal input; see §2.2 for more details. *u*_1_ and *u*_2_ are the *x* and *y* coordinates of the tail tip, respectively. *u*_1_, *u*_2_, *θ*_1_, *θ*_2_, *θ*_3_, *θ*_4_ and *θ*_5_ are generalized coordinates. Note that all generalized coordinates are a function of time. *n_i_* and *t_i_* represent normal and tangential directions of the *i*th link, respectively.

In our model, we assumed an animal body as one-dimensional and discretized it into *N* links. The links are joined by viscoelastic springs to represent the endogenous parameters of the body. The bending stiffness of the springs is represented by *k_i_*, and the internal damping constant is represented by *b_i_*, where *i* represents the joint number such that *i* ∈ [1, *N* − 1]. Particular to the present paper, figure 1 shows the schematic of the body divided into five links *N* = 5 and actuated at one end. The exogeneous parameters are modelled by an anisotropic dry frictional model, constituted by frictional forces in the tangential (equation (2.1)) and the normal (equation (2.2)) directions:

2.1 $${\vec{T}}_{i}=-g\mu_{t}(v_{t,i})m_{i}sgn(v_{t,i}){\hat{t}}_{i}$$

and

2.2 $${\vec{N}}_{i}=-g\mu_{n}(v_{n,i})m_{i}sgn(v_{n,i}){\hat{n}}_{i}.$$

g is the gravitational acceleration constant, $m_{i}$ is the mass of the *i*th link, sgn() is the sign function that gives the sign of normal or tangential velocities, ${\hat{t}}_{i}$ and ${\hat{n}}_{i}$ are unit vectors in the tangential and normal directions, figure 1, $\mu_{t}(v_{t,i})$ is the tangential frictional coefficient as a function of the tangential velocity, and $\mu_{n}(v_{n,i})$ is the normal frictional coefficient as a function of the normal velocity. We consider the frictional coefficients as a function of speed due to the viscoelastic contact between our developed robo-physical model and the substrate (see §2.2); therefore, the trends of the frictional coefficients are found experimentally on different substrates and then approximated by regression analysis (see §3.3 for more details). According to the resistive force theory, to produce forward thrust for lateral undulatory locomotion, the normal frictional coefficient should be higher than the tangential frictional coefficient [27,28]. sgn function in (2.1) and (2.2) is approximated as follows:

2.3 $$\;sgn(\;f\;(x))≃\left[\frac{\;f(x)}{\sqrt{\;f\;{\left(x\right)}^{2}+\epsilon }}\right].$$

Here, the function f(x) can be any function for which we want to determine the sign. Here the parameter ϵ controls the sharpness of the square wave, which is the representation of a sign function. The accuracy of the approximation of sgn increases as ε decreases. We found that to closely match the trend of the experimental speed, on average ε should be set to 10^−3^, especially on a relatively rough substrate, i.e. cloth and cardboard (electronic supplementary material, S1). Physically, the smaller the ε, the sharper the shift in the direction of the frictional force. Since we used viscoelastic material around the wheels (see §2.2), during the locomotion, there is a cyclic transfer of perturbations to the surfaces of the wheels. Because of these disturbances on the viscoelastic material, there is another friction component: the hysteresis component [29]. Furthermore, at the peak of the cycle, when the direction of the frictional force changes, due to the rubber material, the sudden change is limited by the time the material needs to reach its relaxed state before the next cycle. In our model, ε describes this phenomenon, and its value depends on the type of material used and the magnitude of cyclic disturbances around the wheels; in our case, it is observed that ε changes when the substrate is relatively smoother depending upon the cyclic disturbances due to stick and slip. However, for the sake of simplicity and consistency we set the value of ε to 10^−3^.

Equations of motion are formulated by using the lagrangian function as follows:

2.4 $$\frac{d}{dt}\left(\frac{\partial L}{\partial {\vec{\dot{q}}}_{h}}\right)-\frac{\partial L}{\partial {\vec{q}}_{h}}=Q_{h,\mathrm{Dry}}-\frac{\partial R}{\partial {\vec{\dot{q}}}_{h}}.$$

Here, *L* is the lagrangian function, $\vec{q}$ is the vector of generalized coordinates, $Q_{h,\mathrm{Dry}}$ is the generalized force of friction, and *R* is the viscous dissipation energy. Further details of the model can be found in [30].

### 2.2 Physical model

**Figure 2.**
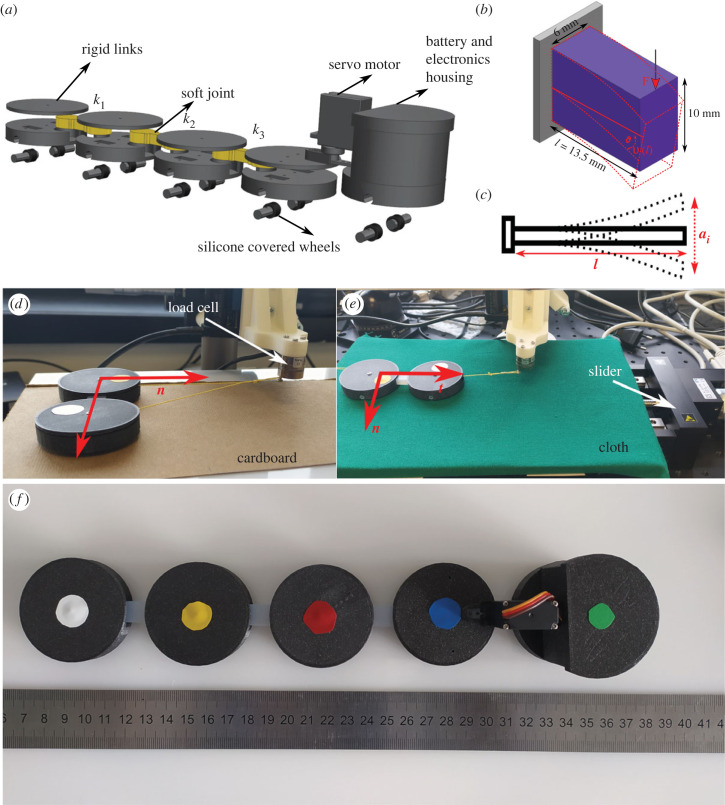
(*a*) Three-dimensional exploded model of the prototype. (*b*) Schematic of bending stiffness test and specimen dimensions. (*c*) Schematic of damping test with the same specimen dimensions as stiffness test. (*d*) Measuring normal frictional coefficient on cardboard substrate. (*e*) Measuring tangential frictional coefficient on cloth substrate. Two modules with a joint are attached to the load cell by a thread. (*f*) Final assembled physical model with markers on each module for tacking. Each segment is 50 mm in diameter. Detailed dimensions and masses are given in electronic supplementary material, S1.

**Figure 3.**
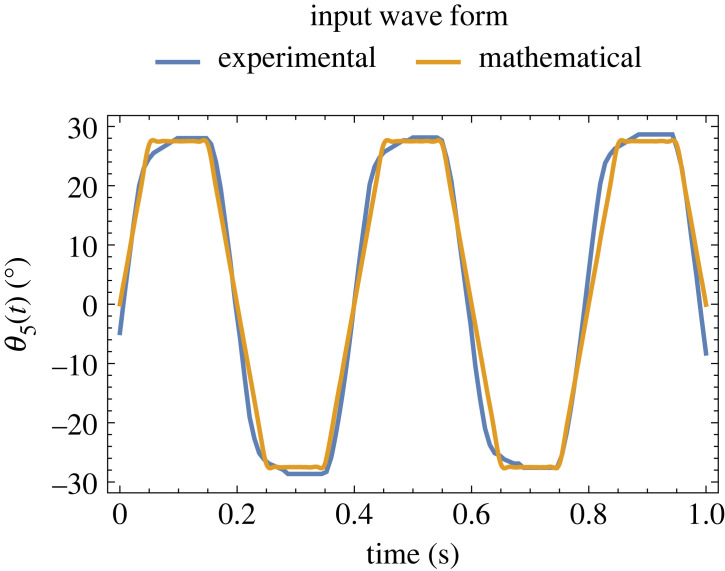
The input waveform obtained from experiments and approximated by a mathematical function.

We implemented a physical system to verify the behaviours achieved in simulation and reiterate in the simulation for behavioural predictions. The system consists of five links. Theoretically, a minimum of three links are required to model friction-driven undulatory locomotion [27,31–33], even though there are no specific criteria for determining the number of links. In our case, it was practical to use five links so that there are at least three passive joints to play with the stiffness distribution. Furthermore, increasing the number of links increases the power utilization [34]. The prototype is fabricated using three-dimensional printed polylactic acid (PLA) modules. The three-dimensional model of the prototype is shown in figure 2*a*. To provide frictional anisotropy, we used two wheels on each module. The wheels consist of a bearing (7 mm external diameter, 4 mm internal diameter and 2.5 mm width) enveloped by a skin of Dragon Skin^TM^ 30 (Smooth-On Inc.). A servo motor (28952, Amewi) is fixed on the head, and a custom electronics and battery supply provide oscillations at desired amplitude and frequency. The angular amplitude and angular frequency of the servo motor, used as the actuator, are kept constant throughout the experiments and are set to (11/72)π rad (27.5°) and 15.7 rad s^−1^ (899.5° s^−1^), respectively. The waveform of the angular frequency is trapezoidal, which is measured by recording and tracking the position of the servo motor horn using a camera and Kinovea software. The waveform is then approximated as defined in electronic supplementary material, S1, and shown in figure 3. The total length and mass of the physical model are 287 mm and 132 g, respectively. Masses and lengths of individual links are listed in electronic supplementary material, S1. Joints are prototyped with five materials of different stiffness: Dragon Skin^TM^ 10, Dragon Skin^TM^ 30 (both from Smooth-On Inc.), polydimethylsiloxane (PDMS, Sylgard 184, Dow), Elastic 50A and Flexible 80A (both from Formlabs). Joints made of Dragon Skin^TM^ 10, Dragon Skin^TM^ 30 and PDMS materials are cast from their constituent parts at room temperature. Joints with Elastic 50A and Flexible 80A materials are fabricated by stereolithography (SLA) three-dimensional printing with a layer thickness of 0.1 mm.

### 2.3 Experimental characterization

The equivalent bending stiffness of the joints is calculated experimentally by measuring their deflections and force responses (using a Z005 Universal Testing Machine, ZwickRoell) in a cantilever configuration, as shown in figure 2*b*. Similarly, damping constants are calculated using the logarithmic decrement method [35], and tests are performed in a cantilever beam configuration. The schematic is shown in figure 2*c*. Videos of the cantilever beam tests are recorded with a high-speed camera (Phantom Micro C110) at a frame rate of 2300 f s^−1^ to determine the amplitude dissipation over time. To emulate different environments, we used four substrates: polyoxymethylene copolymer (POMC), plastic panel, cardboard, and cloth. As shown in figure 2*d*,*e*, longitudinal and lateral frictional constants are found on these substrates. In friction tests, we utilized two modules of the physical model connected by a joint. The modules are attached to a load cell with a nearly inextensible nylon thread (Nano17 SI-12-0.12, ATI Industrial Automation). The substrate is then moved at constant speeds by motorized micro-translation stages (M-414.2PD, Physik Instrumente GmbH). Finally, the behaviour of the physical model on different substrates with different joint stiffnesses is captured by tracking markers on the modules, as shown in figure 2*f*. The videos are captured by a Nikon D7500 camera and post-processed using the free, open-source software Kinovea^1^.

## 3. Results

### 3.1 Equivalent bending stiffness of joints

Equivalent bending stiffness, *k*, is calculated using equation (3.1). The proof is provided in electronic supplementary material, S1:

3.1 $$k=\frac{Fl^{2}}{3\upsilon (l)}.$$

**Figure 4.**
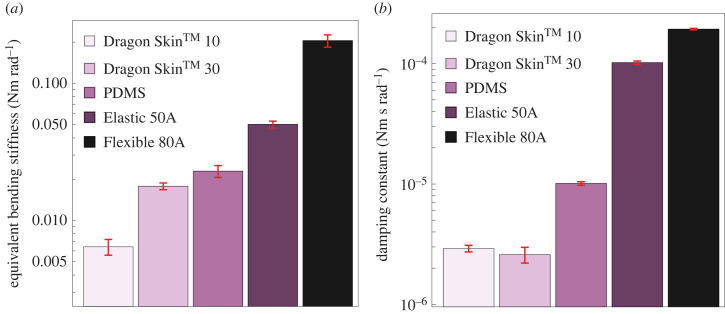
(*a*) Measured equivalent bending stiffness of various materials used as a joint material. (*b*) Measured damping constants.

Here *F* is the force applied and recorded by load cell, *l* is the length of the specimen and υ(l) is the deflection recorded from the universal testing machine under the action of force at a distance *l* from the fixed end. The calculated equivalent bending stiffness of different materials is shown in figure 4*a*.

### 3.2 Damping constants of joints

Damping constants are calculated using the logarithmic decrement method according to (3.2) [35]. A derivation is provided in electronic supplementary material, S1:

3.2 $$b=2\zeta \sqrt{kI}.$$

In equation (3.2), *I* is the moment of inertia of a rectangular cross-section area of a beam, calculated as *ml*^2^/3; here, *m* is the mass of the specimen. *ζ* is the damping ratio calculated by the logarithmic decrement method as follows:

3.3 $$\zeta =\frac{\delta }{\sqrt{4\pi^{2}+\delta^{2}}}.$$

Here in equation (3.3), *δ* is measured experimentally according to the following equation:

3.4 $$\delta =\frac{1}{n}ln\left(\frac{a_{1}}{a_{n+1}}\right).$$

Equations (3.3) and (3.4) are taken from [35], where *a*_1_ is the peak-to-peak distance of oscillations at time, *t*, and *a*_*n* + 1_ is the peak-to-peak distance after *n* oscillations. Calculated damping constants are shown in figure 4*b*.

### 3.3 Friction tests

**Figure 5.**
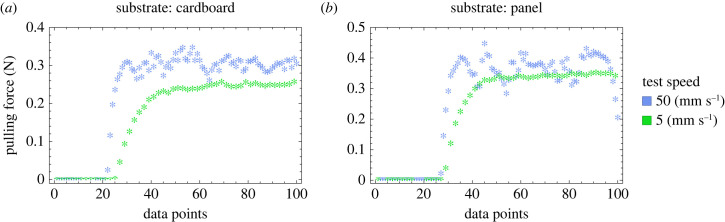
The raw data from the force measurements in the experiments to calculate the normal frictional coefficient. At higher speeds the collected data are more scattered. This shows that stick and slip occur more frequently as the speed increases, especially on smoother substrates. (*a*) Cardboard substrate. (*b*) Panel substrate.

In our case, the classical model of Coulomb friction posed some limitations due to a non-uniform sliding of the system also dictated by a friction coefficient velocity dependence. This phenomenon can occur because of the viscoelastic material used around the wheels. This issue is covered by finding the trend of average frictional coefficients at various speeds. The average dynamic normal and tangential frictional coefficients are measured according to the formula *μ* = *F*_p_/*W* at various speeds to determine their speed-dependent trends. *F*_p_ is the pulling force calculated by the load cell, and *W* is the weight of the specimen. Figure 5 displays the increasing effect of stick and slip with speed by comparing the raw measurement of the pulling force for both cardboard and panel substrates used in the tests to calculate the normal frictional coefficients. Similar behaviour is also observed for other substrates. The phenomenon of stick and slip is predominantly observed in the normal direction because of the constraint provided by wheels in that direction.

Further assumptions of the frictional model include (1) no deformation and asymmetric normal pressure distribution in the wheels, (2) there is no temperature change during the motion at the contact area and its surroundings, and (3) friction is independent of the contact area between the body and the ground.

**Figure 6.**
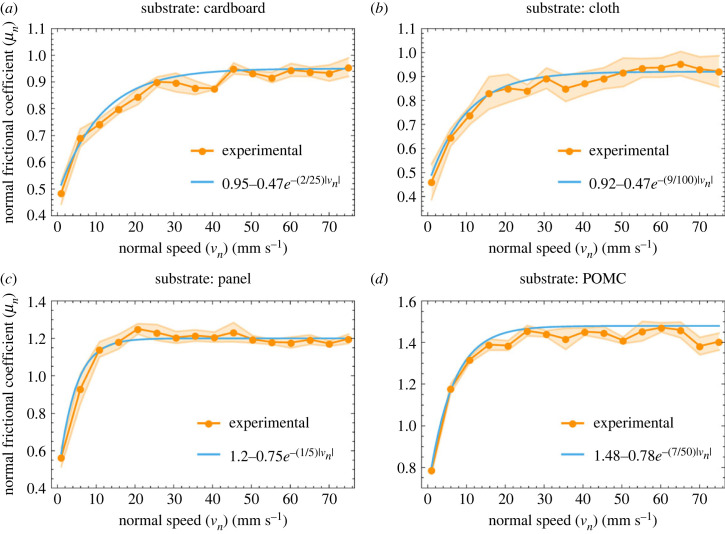
Normal frictional coefficient on various substrates at different speeds. (*a*) Cardboard. (*b*) Cloth. (*c*) Panel. (*d*) POMC.

**Figure 7.**
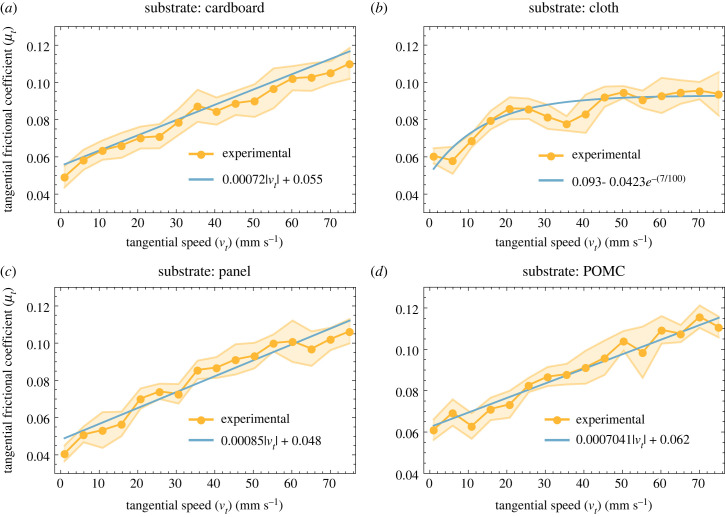
Tangential frictional coefficient on various substrates at different speeds. (*a*) Cardboard. (*b*) Cloth. (*c*) Panel. (*d*) POMC.

The results of the normal frictional tests are shown in figure 6, and the tangential frictional coefficients on various substrates are shown in figure 7. The trend of the normal frictional coefficient is estimated by exponential plateau curve equations, as shown in figure 6. The average value of the normal frictional coefficient ratio stabilizes after certain speeds. The value of the frictional coefficient higher than 1 shows the dominance of the sticking phenomenon. While in the cases where the frictional coefficient is lower than 1, slippage is the dominant phenomenon. On cardboard and cloth, the normal frictional coefficient ratio did not reach 1 because these substrates are less smooth than the panel and POMC substrates; consequently, they offered less affinity to adhesion. Unlike the normal frictional coefficient, the tangential frictional coefficient reaches an equilibrium state only in the case of cloth. In the other cases, the tangential frictional coefficient increases with speed (figure 7). The frictional coefficient ratio (*μ*_n_/*μ*_t_) on these substrates is given in electronic supplementary material, S1.

### 3.4 Comparison between mathematical and physical models

**Figure 8.**
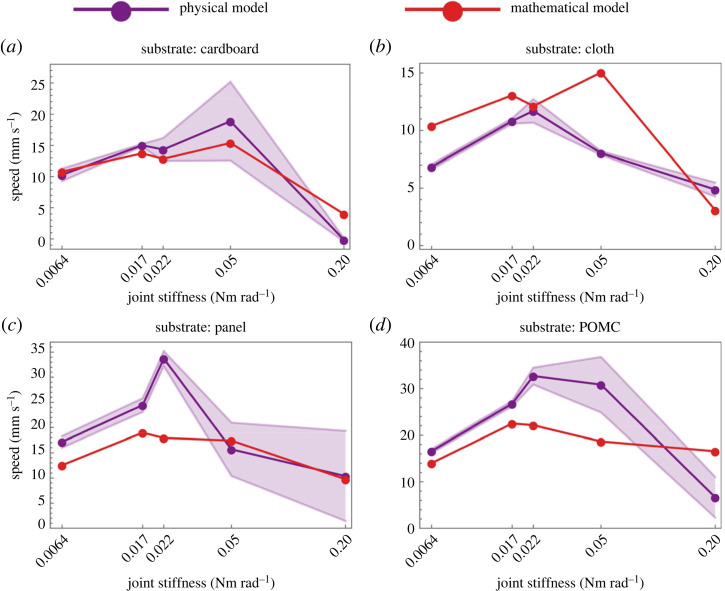
Comparison between mathematical and physical speeds obtained on various substrates and using different stiffnesses. The effect of the stiffness and environment can be seen in optimizing the speed. (*a*) On cardboard, results are quantitatively and qualitatively comparable. (*b*) On cloth, results are quantitatively comparable. (*c*) On panel, results are quantitatively comparable. (*d*) On POMC, results are quantitatively comparable.

**Figure 9.**
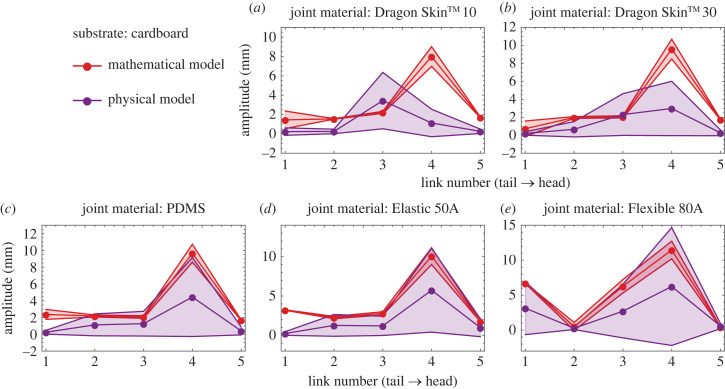
Comparison between experimentally and theoretically observed amplitudes of the centre of each link on cardboard substrate. (*a*) Joint material is Dragon Skin^TM^ 10. (*b*) Joint material is Dragon Skin^TM^ 30. (*c*) Joint material is PDMS. (*d*) Joint material is Elastic 50A. (*e*) Joint material is Flexible 80A.

After evaluating all required input parameters, we ran the simulations based on a mathematical model and compared the speeds at different joint stiffnesses and on different substrates. Overall, the physical and mathematical models showed significant agreement, as shown in figure 8*a–d*. The mathematical model quantitatively captured the generalized trend within a factor of 2. A side by side comparison of the physical system along with trajectories is given in electronic supplementary material, video S1. Physically and mathematically, it can be witnessed that the speed optimization depends on the environment and joint stiffness. Experimentally, the maximum speed occurs when joints of Elastic 50A, PDMS, PDMS and Elastic 50A were used on cardboard, cloth, panel and POMC, respectively (figure 8*a*–*d*). The comparison between experiments and simulations is shown in electronic supplementary material, video S1. It is observed that the amplitude of the tail increases as the stiffness of the joints increases, while the speed increases as the stiffness increases and then decreases as the stiffness increases further. For cardboard, the trend of the amplitudes of each joint for various joint stiffnesses is shown in figure 9. A similar trend is observed for other substrates (see electronic supplementary material, S1).

When considering the origin of the disparity between the mathematical and physical models, it is noteworthy that hysteresis is one of the contributing factors. The hysteresis acting on the wheels arises due to the cyclic loads and the material properties of the wheel coverings (Dragon Skin 30^TM^). Passive deformation of the wheel coverings due to the cyclic load induces a time lag in the change of friction direction. In our mathematical model, the hysteresis should be captured by the ε parameter (equation (2.3)) because it controls the smoothness of the square wave, a sign function to obtain the direction of the friction force. The smaller the value of ε, the more accurate the square wave, and the sharper will be the change in the direction of friction; therefore, the smaller will be the hysteresis. Physically the hysteresis component of the friction depends on the internal friction of the material due to cyclic loads [29], and it has been observed that at higher speeds, the transfer of cyclic disturbances increases to the wheels on smooth substrates, i.e. panel and POMC; consequently, it increases the deformation of the wheel coverings and, therefore, the hysteresis. That is why it is observed that the value of ε increases at higher speeds on the panel and POMC substrates to match the physical and mathematical results, as shown in electronic supplementary material, S1, figure S5. However, in many instances, the value of ε was observed to be 10^−3^, which is why we used only one value in all simulations for simplicity and continuity. Another source of discrepancy is the contact loss during locomotion which is inherent in the physical implementation due to manufacturing tolerances. Especially on the smoother substrates (panel and POMC) at higher stiffnesses (figure 8*c*,*d*), we see more standard deviation resulting from vibrations because of the oscillations and random contact losses with the ground. At higher stiffnesses, contact losses increase because of the induced rigidity in the system, as can also be seen in the cardboard case at 0.05 Nm rad^−1^ stiffness (figure 8*a*). While on a relatively rough substrate (cloth), the standard deviation remained small (figure 8*b*). In addition, other possible sources of error include simplified assumptions of one-dimensional geometry, inextensible joints and neglect of out-of-plane dynamics of locomotion.

### 3.5 Optimization based on stiffness distribution

**Figure 10.**
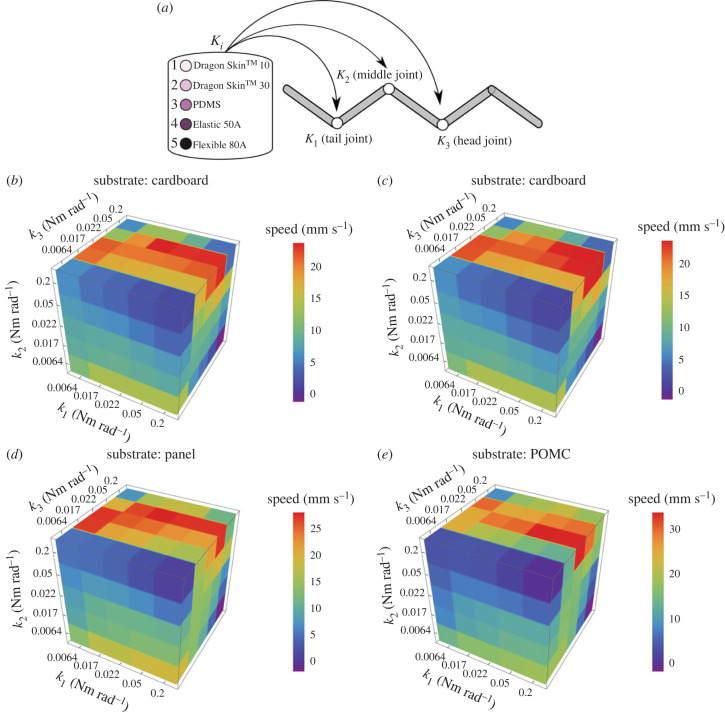
(*a*) Schematic of a five-link system with three joints where five different joint stiffnesses are tried to determine the optimum combination. The different stiffnesses of the materials utilized in this research are labeled from 1 to 5, where 1 represents the equivalent bending stiffness of Dragon Skin 10^TM^, which is the lowest in our collection, and 5 represents the equivalent bending stiffness of Flexible 80A, which is the highest in our collection. (*b*–*e*) Simulated results of different stiffness combinations on different substrates. Each coloured block represents a speed at a specific stiffness combination. Here, equivalent bending stiffnesses of 0.0064 Nm rad^−1^, 0.017 Nm rad^−1^, 0.02 Nm rad^−1^, 0.05 Nm rad^−1^ and 0.2 Nm rad^−1^ are labelled 1, 2, 3, 4 and 5, respectively. The general qualitative trend can be formulated as *k*_1_ ≤ *k*_2_ > *k*_3_ for all substrates by observing optimum combinations. (*b*) The optimum stiffness combination suggested by the simulation in the cardboard case is (3, 5, 4) as (*k*_1_, *k*_2_, *k*_3_), respectively. According to the mathematical model, the maximum speed is 23.78 mm s^−1^, and the speed found experimentally is 31.93 ± 0.34 mm s^−1^. There is approximately 69% increase from the maximum value when all joints have the same stiffness, i.e. (4, 4, 4) in this case (figure 8). (*c*) The optimum stiffness combination suggested by simulation in the case of cloth is (5, 5, 3) as (*k*_1_, *k*_2_, *k*_3_). According to the mathematical model, the maximum speed is 23.6 mm s^−1^. The speed found experimentally is 33.3 ± 0.5 mm s^−1^ which represents approximately 184% increase compared to the maximum value when all joints have the same stiffness, i.e. (3, 3, 3) in this case (figure 8). (*d*) The optimum stiffness combination suggested by simulation in the panel case is (3, 5, 4) as (*k*_1_, *k*_2_, *k*_3_). According to the mathematical model, the maximum speed is 28.20 mm s^−1^, and the speed found experimentally is 34.3 ± 1.88 mm s^−1^. This represents an increase of approximately 2% compared to the maximum when all joints have the same stiffness, i.e. is (3, 3, 3) in this case (figure 8). (*e*) The optimum stiffness combination suggested by simulation in the case of POMC is (4, 5, 3) as (*k*_1_, *k*_2_, *k*_3_). According to the mathematical model, the maximum speed is 33.74 mm s^−1^, and the speed found experimentally is 38.4 ± 4.6 mm s^−1^. This represents an increase of approximately 17% compared to the maximum when all joints have the same stiffness, i.e. is (3, 3, 3) in this case (figure 8).

**Figure 11.**
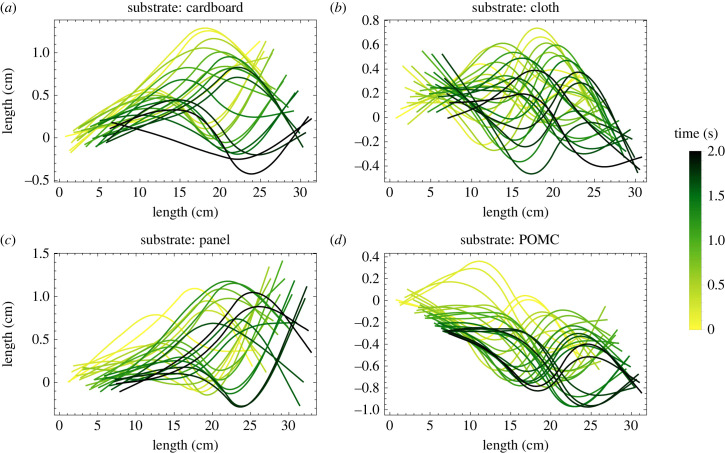
Position of the centreline of the robot is constructed at various time intervals by joining the positions of the tracked points, as mentioned in figure 2*f*, using second-order polynomial. The period of the input wave is 0.4 s. (*a*) 5 snapshots per period of the oscillations are provided for the cardboard substrate. Data are taken from the experiment of the optimum stiffness distribution of (3, 5, 4). (*b*) 6 snapshots per period of the oscillations are provided for the cloth substrate. Data are taken from the experiment of the optimum stiffness distribution of (5, 5, 3). (*c*) 5 snapshots per period of the oscillations are provided for the panel substrate. Data are taken from the experiment of the optimum stiffness distribution of (3, 5, 4). (*d*) 6 snapshots per period of the oscillations are provided for the POMC substrate. Data are taken from the experiment of the optimum stiffness distribution of (4, 5, 3).

We additionally explored the relationship between speed and stiffness distribution among the joints. Five different stiffnesses are available and combined over three joints, resulting in 125 combinations for each substrate, shown in figure 10*a*. To simplify the presentation of different stiffness combinations, we defined a convention of naming different joint materials according to their ascending order of stiffness from 1 to 5, as shown in figure 10*a*. After running simulations for all stiffness combinations on various substrates, we found that qualitatively the highest speed is achieved when the stiffness of the middle joint is higher than that of the head joint, and subsequently decreases or remains constant towards the tail (*k*_1_ ≤ *k*_2_ > *k*_3_), as shown in figure 10*b–e*. By following the combinations suggested by the simulations on the physical model, we obtained approximately 69%, 184%, 2% and 17% higher speeds on cardboard, cloth, panel and POMC substrates, respectively. The percentage increase is calculated by comparing the experimental speeds of the optimal cases obtained from figure 10 and figure 8. Figure 11 shows snapshots of the robot's centreline for the optimal combinations of stiffness distributions across various substrates. These images provide a visual representation of the temporal evolution of undulatory locomotion. Furthermore, the trend of stiffness combination changes on different substrates (figure 10*b–e*). It is inferred that the stiffness distribution could differ qualitatively and quantitatively in different environments. To consolidate the numerical results, we tested the false optimality condition, deduced from the simulations, on the panel substrate. The false condition defines that the bending stiffness at the joints must be *k*_1_ > *k*_2_ ≤ *k*_3_. Based on this, 40 different combinations are tested on the panel substrate. The tested combinations and their results are listed in electronic supplementary material, S2. In all cases, the speed is less than 34.3 ± 1.88 mm s^−1^, corresponding to the stiffness combination (*k*_1_,*k*_2_,*k*_3_ = 3,5,4) as suggested by the simulations. In addition, after testing the validity of the optimality condition, we have experimentally found the optimum stiffness combination using the qualitative result of simulations producing the criteria *k*_1_ ≤ *k*_2_ > *k*_3_. In our experiments, we eliminated the outliers for *k*_3_ = 1,5. The sliced planes of figure 10*b–e* in electronic supplementary material, S1, reveal that at *k*_3_ = 1, the stiffness is insufficient to achieve the maximum speed. Conversely, when *k*_3_ = 5, no combination adheres to the inferred law of optimal stiffness since 5 is the highest stiffness available, and the condition *k*_2_ > *k*_3_ cannot be fulfilled. Hence, a total of 26 different combinations are tested on each substrate. The results of optimum combinations found experimentally are listed in table 1, and to see the experiments of optimum cases, see electronic supplementary material, video S2.

**Table 1.** Comparison of optimum stiffness distribution found in simulations and experimentally using the qualitative results suggested by simulations.

substrate	simulation			experiments		
	combinations (*k*_1_, *k*_2_, *k*_3_)	percentage increase	speed (mm s^−1^)	combinations (*k*_1_, *k*_2_, *k*_3_)	percentage increase	speed (mm s^−1^)
cardboard	(3,5,4)	69	31.93 ± 0.34	(3,4,2)	71	32.38 ± 0.21
cloth	(5,5,3)	184	33.3 ± 0.5	(5,5,3)	184	33.3 ± 0.5
panel	(3,5,4)	2	34.30 ± 1.88	(4,5,2)	31	44.5 ± 0.15
POMC	(4,5,3)	17	38.4 ± 4.6	(3,5,4)	51	49.65 ± 0.21

Based on both simulations and experimental results, it can be deduced that the environment strongly affects locomotion gaits and speeds. The hysteresis component is likely causing quantitative differences between simulation and experiment, which is assumed not to vary for each joint in our simulations. Nevertheless, we can define the stiffness distribution law for each environment to maximize the speed of locomotion.

### 3.6 The influence of input frequencies

At resonance, when the input frequency matches with the natural frequency, in a fluidic environment the speed of undulatory locomotion increases [20,36]. Contrarily, in [24] for dry frictional environment, it is found that at resonance, the speed of undulatory locomotion decreases and the power increases. More recently, in [37], for viscous friction, the resonance frequency is defined as the frequency at which the speed of a system is maximized with minimum actuation effort for undulatory locomotion. In this section we study the correlation between the input frequency and the resulting speed. We then subsequently relate these findings to the optimization process based on stiffness, as presented in figure 8 and figure 10. We limited our input frequencies to the range of 1–30 rad s^−1^ for uniform stiffness distribution and 1–50 rad s^−1^ for non-uniform stiffness distribution. These limits are set based on the findings of a previous study [36], which demonstrated that higher forcing frequencies induce deformation modes that hinders forward motion. Hence, lower deformation modes are typically observed in biological systems due to their effectiveness in generating propulsion.

**Figure 12.**
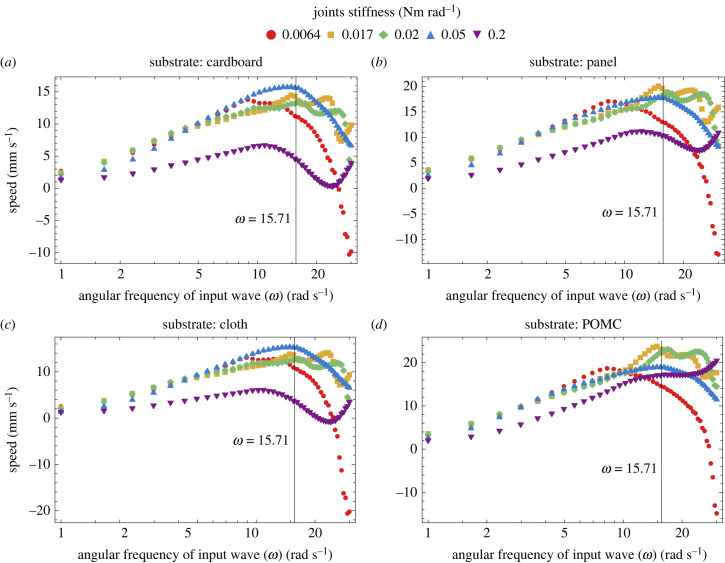
Simulations are run at various input frequencies ranging from 1 to 30 rad s^−1^ for all substrates to find the peaks of maximum speed. The body has a uniform stiffness distribution across its joints. The amplitude and waveform of the input wave are the same as that of other simulations performed in this article. (*a*) The maximum speed occurred at 8.92 ± 0.6 rad s^−1^ for 0.0064 Nm rad^−1^; at 14.86 ± 0.6 rad s^−1^ and 22.78 ± 0.6 rad s^−1^ for 0.017 Nm rad^−1^; at 9.58 ± 0.6 rad s^−1^, 16.18 ± 0.6 rad s^−1^ and 26.08 ± 0.6 rad s^−1^ for 0.02 Nm rad^−1^; at 14.86 ± 0.6 rad s^−1^ for 0.05 Nm rad^−1^; at 10.9 ± 0.6 rad s^−1^ for 0.2 Nm rad^−1^. (*b*) The maximum speed occurred at 8.26 ± 0.6 rad s^−1^ for 0.0064 Nm rad^−1^; at 14.86 ± 0.6 rad s^−1^ and 22.12 ± 0.6 rad s^−1^ for 0.017 Nm rad^−1^; at 9.6 ± 0.6 rad s^−1^, 16.8 ± 0.6 rad s^−1^ and 24.6 ± 0.6 rad s^−1^ for 0.02 Nm rad^−1^; at 14.86 ± 0.6 rad s^−1^ for 0.05 Nm rad^−1^; at 12.22 ± 0.6 rad s^−1^ for 0.2 Nm rad^−1^. (*c*) The maximum speed occurred at 8.92 ± 0.6 rad s^−1^ for 0.0064 Nm rad^−1^; at 14.86 ± 0.6 rad s^−1^ and 23.44 ± 0.6 rad s^−1^ for 0.017 Nm rad^−1^; at 9.58 ± 0.6 rad s^−1^, 16.18 ± 0.6 rad s^−1^ and 26.08 ± 0.6 rad s^−1^ for 0.02 Nm rad^−1^; at 14.86 ± 0.6 rad s^−1^ for 0.05 Nm rad^−1^; at 10.9 ± 0.6 rad s^−1^ for 0.2 Nm rad^−1^. (*d*) The maximum speed occurred at 8.26 ± 0.6 rad s^−1^ for 0.0064 Nm rad^−1^; at 14.86 ± 0.6 rad s^−1^, 20.14 ± 0.6 rad s^−1^ and 28.72 ± 0.6 rad s^−1^ for 0.017 Nm rad^−1^; at 16.84 ± 0.6 rad s^−1^ and 23.44 ± 0.6 rad s^−1^ for 0.02 Nm rad^−1^; at 14.86 ± 0.6 rad s^−1^ for 0.05 Nm rad^−1^.

**Figure 13.**
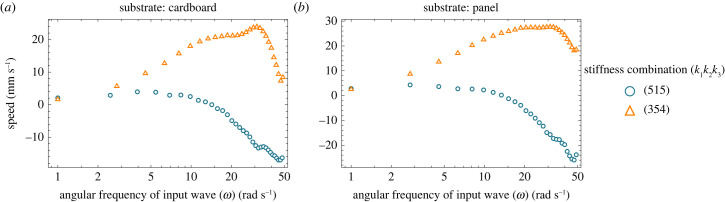
(*a*,*b*) Simulations are performed at various input frequencies ranging from 1 to 50 rad s^−1^ for cardboard and panel substrates to find the trend of the speed for (5,1,5) and (3,5,4) cases of stiffness distribution, respectively. Convention used in the naming of stiffnesses distribution is presented in figure 10*a*.

Figure 12*a*–*d* shows the speed peaks achieved at different actuation frequencies for different joint stiffness values on different substrates. While comparing the results of the speed peaks across substrates, it can be seen that the effect of a different environment on defining the optimum input frequency is negligible. By contrast, the optimum input frequency depends on the stiffness of the body (figure 12*a*–*d*). As also elaborated in the detailed study of the resonance of undulatory locomotion in [37], that resonance frequency depends only on body stiffness and inertia of a body and that resonance frequency enhances the speed. In our case, the body's inertia remained constant and only the stiffness of the body and the friction of the environment changed. Therefore, following [37] we can say that the peaks of the speeds observed in figure 12 are at resonance frequencies. Furthermore, our results show that the environment plays an important role in defining the height of the peak (the maximum locomotion speed), which makes certain stiffness values suitable for certain environments. For example, on cardboard, the joint stiffness of 0.05 Nm rad^−1^ provides the highest locomotion speed. This is consistent with the results of figure 8*a*, where we found the stiffness of 0.05 Nm rad^−1^ to be the best for cardboard both experimentally and analytically. Whereas, in the case of panel and POMC substrates, a joint stiffness of 0.017 Nm rad^−1^ has the highest speed, and in the case of cloth a joint stiffness of 0.05 Nm rad^−1^ has the highest speed in the input frequency range of 1–30 rad s^−1^. We also investigated the response to the input frequency when the joint distribution is not uniform for cardboard and panel substrates (figure 13*a*,*b*). Stiffness distribution of (3,5,4) among joints 1, 2 and 3 is selected according to the law of optimum stiffness distribution (*k*_1_ ≤ *k*_2_ > *k*_3_) as found in §3.5, and stiffness distribution of (5,1,5) is selected contrarily to the case of optimum stiffness distribution law. These figures highlight the two main advantages of applying the law found for stiffness distribution: firstly, to enhance the speed; secondly, it increases the range of the effective input frequencies as compared to figure 12 when uniform stiffness is employed.

### 3.7 Locomotion characterization based on Froud number

The ratio of inertial forces to other relevant forces plays a pivotal role in characterizing gaits. In terrestrial locomotion, this ratio is called the Froude number [38]. Animals with equivalent Froude numbers walk and run in a dynamically similar manner [39]. In friction-dominant locomotion, the Froude number can be defined as *F*_r_ = *λ*/(*μ*_n,max_*τ*^2^*g*) [40]. Here *λ* is the stride length which we are taking as the average wavelength of the trajectories traced by the physical model segments at their tracking points. *μ*_n,max_ is the maximum calculated normal frictional coefficient on each substrate, since the normal frictional coefficient is the component of the friction forces acting on the body responsible for the propulsion [41]. *τ* is the period of the oscillations, in our case equal to 0.4 s, and *g* is the gravitational acceleration constant. The lower Froude number indicates the dominance of frictional forces. Our calculated range of Froude number is approximately 0.004–0.01 (electronic supplementary material, S2), defining the friction-dominant nature of the locomotion.

## 4. Discussion

We investigated the effects of passive stiffness and environment on lateral undulatory locomotion by comparing mathematical and physical models to determine the performance optimization criteria. Our findings suggest a strong correlation between the resultant locomotion and the surrounding environment and body properties. We observed that changing stiffness affects locomotion in an environment, and stiffness has different responses in different environments. These relationships are evident in some living organisms. For example, eels modulate their body stiffness to achieve different performances in the same environment [4,42]. They increase their speed by engaging more muscles and increasing their body stiffness. As also predicted for sunfish, stiffness can be doubled to increase the speed [43]. A larval zebrafish-inspired robot also showed the significance of right stiffness for locomotion [44]. Furthermore, it is also reported how changing environments influence locomotion, e.g. in *Caenorhabditis elegans* [3,21,45,46] speed in low-viscous fluids is faster than in more viscous environments [10,22].

Experimental tests and simulations show that the tail amplitude increases as body stiffness increases, regardless of whether speed is increased or decreased. When animals change their gait from swimming to crawling, it is either because of the passive interaction with the environment [3,47] or because of the active increase of speed [4], with consequent change of wave kinematics. As in the case of swimming, the body wave amplitude increases from anterior to posterior, whereas in the case of crawling gait, the body wave amplitude either remains the same or decreases from anterior to posterior. Our results elucidate the functional role of the environment and passive body stiffness in body wave kinematics.

The distribution of body stiffness plays an essential role in the performance of limbless animals. In sunfish, flexural stiffness increases and decreases from head to tail [43]. Typically, the highest stiffness is observed to be three orders of magnitude greater than the lowest stiffness. Computational results on lamprey locomotion have showed the importance of tail flexibility [20]. In particular, as tail stiffness increases, wakes become less coherent, and speed performance decreases. However, in [44], it is found that non-uniform body stiffness does not lead to more satisfactory performance than a uniform stiffness distribution. Our analysis reveals that the physical model performs better when the stiffness of the middle joint is higher than the head joint. Furthermore, our results suggest that when the environment changes, the quantitative requirement of stiffness distribution changes; however, the qualitative trend of the stiffness distribution remains preserved.

Resonance frequency is another determining factor in the dynamics of undulatory locomotion. Changing the stiffness of the joints, while keeping other body parameters constant, sets the natural frequency of the body regardless of the environment. This defines different preferences of stiffness in different environments. Biological evidence for this phenomenon can be found. For example, in animals, central pattern generators regulate the rhythmic movements of the body at its natural frequency in coordination with the feedback from the environment [48]. Furthermore, we also know that animals can modulate their body stiffness through different muscle engagements. For example, eels have been observed to recruit more muscles under certain circumstances to increase speed [4,42]. By manipulating the stiffness distribution along the body, our investigation reveals that the body's response can be optimized over a broad range of input frequencies, rendering it less susceptible to frequency sensitivity and augmenting its velocity.

The characterization of the locomotion based on the Froude number showed the dominance of frictional forces over inertial forces. Furthermore, a lower Froude number means a shorter time taken to reach the steady state. Our calculated range of the Froude numbers is consistent with those obtained for snake-slithering locomotion [40,41].

The discrepancy between physical and mathematical models can be attributed to modelling simplifications such as uniform mass distribution, rigid links, and reduced geometric dimensionality. It is found that accurate modelling of the nature of the interacting bodies plays an essential role in the resulting locomotion. In addition, out-of-plane motion due to random vibrations, instantaneous contact loss and manufacturing tolerances can also introduce discrepancies.

## 5. Conclusion

We analysed and found a correlation between body stiffness and the environment for limbless undulatory locomotion in a dry friction environment. Our mathematical results are in agreement with physical experiments. The results suggest that the interdependence between passive body stiffness and environment can be exploited to build efficient undulatory robots that need to operate in specific environments. Furthermore, the speed of undulatory locomotion can be improved by utilizing a non-uniform stiffness distribution along the length of the body. The stiffness distribution can be arranged in either an ascending–descending or ascending–plateau order. Future work includes exploiting body material properties and patterns instead of wheels as a frictional interface with the environment and employing learning algorithms to aid understanding gait responses over cluttered environments.

## Data Availability

The data are provided in the electronic supplementary material [49].

## Endnote

1 See https://www.kinovea.org/.
